# Detecting germline BAP1 mutations in patients with peritoneal mesothelioma: benefits to patient and family members

**DOI:** 10.1186/s12967-018-1559-7

**Published:** 2018-07-13

**Authors:** Muaiad Kittaneh, Charles Berkelhammer

**Affiliations:** 1 Loyola University, 15300 West Avenue, Orland Park, IL 60462 USA; 2 University of Illinois, 9921 Southwest Highway, Oak Lawn, IL 60453 USA

**Keywords:** BAP1, Germline, Familial, Mesothelioma, Melanoma, Screening, Early detection

## Abstract

Germline mutations in the BRCA-1 associated tumor protein 1 (BAP1) increase susceptibility to mesothelioma and other cancers. We describe a patient with a family history of peritoneal mesothelioma, who developed malignant peritoneal mesothelioma at age 45 in the absence of known asbestos exposure. These findings lead us to hypothesize that the mesothelioma occurred in the setting of germline a BAP1 mutation. This was confirmed by genetic testing. The subsequent therapeutic choices for the patient and testing of at-risk family members highlight the importance of recognizing this genetic syndrome and screening for individuals at high risk.

## Background

Germline mutations in the BRCA-1 associated tumor protein 1 (BAP1) increase susceptibility to mesothelioma, uveal and cutaneous melanomas, renal cell carcinomas, basal cell and squamous cell carcinomas, as well as, although less frequently, to other cancer types [1–6]. This condition, named as the “BAP1 cancer syndrome” [5–7] is transmitted in a Mendelian fashion. Thus, about 50% of the progeny of carriers of BAP1 mutations are expected to inherit the mutation. Initially described, in two families from Louisiana to Wisconsin [8], BAP1 cancer syndrome has now been described in multiple families across the world [2, 7, 9, 10]. Among about 100 BAP1 families reported in the literature so far, a very large multi-generation family originating from a couple who immigrated to the US in the early 1700’s from Germany has been described in the US [9]. Similarly to patients with the Li-Fraumeni syndrome, BAP1 mutations are highly penetrant, and so far most carriers of BAP1 mutations have developed one or more malignancies during their lifetime [11]. The biological reasons that account for the powerful tumor suppressor activity of BAP1, a deubiquitylating enzyme, have been recently elucidated [12–17]. It has been shown that BAP1 localizes both in the nucleus and in the cytoplasm. In the nucleus BAP1 regulates DNA repair by homologous recombination [12, 13]. In the cytoplasm, BAP1 deubiquitinases and thus stabilizes IP3R3, the channel that allows calcium (Ca^2+^) to leave the endoplasmic reticulum—where Ca^2+^ is normally stored-, and reach the mitochondria, where Ca^2+^ regulates aerobic respiration and programmed cell death/apoptosis [14, 15]. Cells in carriers of germline BAP1 mutations have reduced levels of BAP1, about 50% than normal as these cells have only one normal BAP1 allele [14]. Human cells derived from carriers of germline BAP1 mutations showed reduced ability to repair DNA by homologous recombination and to execute apoptosis following exposure to asbestos, ultraviolet light and irradiation [14]. Moreover these same cells derive energy largely through aerobic glycolysis (Warburg effect) probably as a consequence of the reduced mitochondrial Ca^2+^ levels which are required for the activity of several enzymes that regulate the Kreb’s cycle [15]. The consequence of these alterations are that when cells carrying heterozygous mutations are exposed to environmental carcinogens, they accumulate but cannot properly repair DNA mutations (because of the reduced levels of nuclear BAP1) [14]. Under normal circumstances accumulation of DNA mutations triggers apoptosis, but BAP1 mutant cells have impaired apoptosis because of the reduced Ca^2+^ mitochondrial levels [14]. Therefore these cells accumulate genetic alterations that are passed to daughter cells that are prone to malignant transformation [14]. When these cells become transformed, they are already capable of invading nearby tissues and of growing in a hypoxic environment since they derive energy largely through aerobic glycolysis [15]. These mechanisms account for the high rate of cancer, especially those cancers caused by environmental carcinogens in carriers of germline BAP1 mutations [16]. Cancers that develop in carriers of germline BAP1 mutations are less aggressive, have a better prognosis and improved survival [11]. The reasons for the improved survival are presently unclear. However, early detection, resulting from close monitoring of affected families is a likely factor [7]. Detecting BAP1 mutations in appropriate cancer patients is important since management may be affected. Family members can be screened for the same mutation. If a BAP1 mutation is present, then screening and early detection of BAP1-associated cancers can be undertaken. Other preventative measures can be instituted including minimizing exposure to ultraviolet light and asbestos.

Here we describe a female patient with a strong family history of peritoneal mesothelioma, who developed malignant peritoneal mesothelioma at a young age in the absence of known asbestos exposure.

## Main text

### Case presentation

**Fig. 1:**
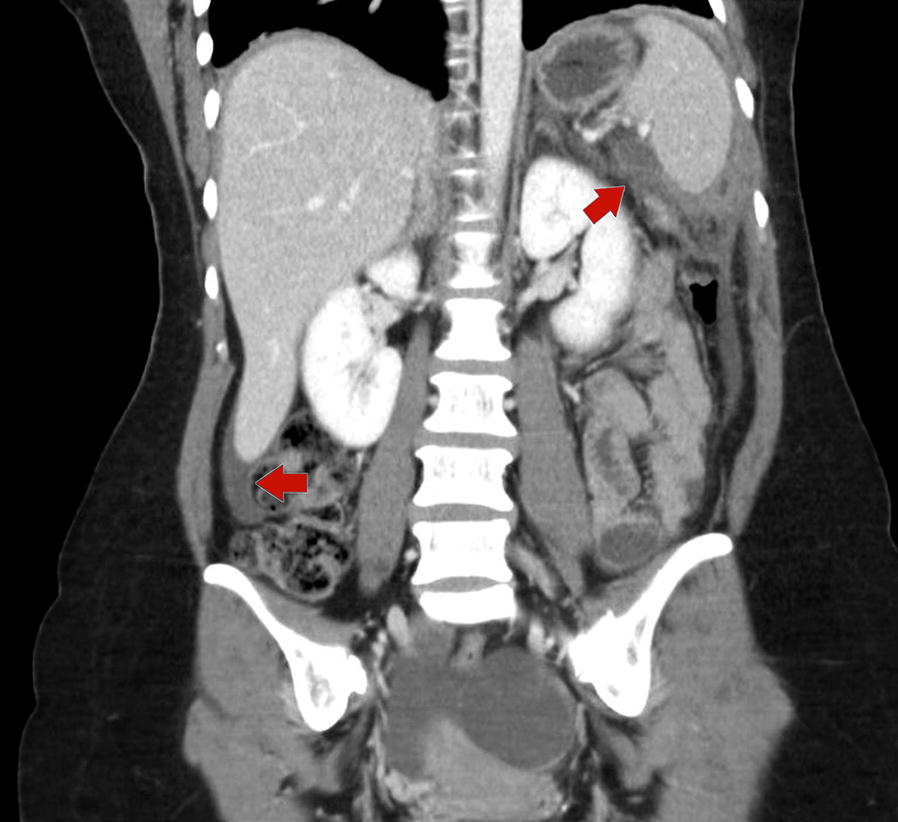
Computed tomography of the abdomen demonstrating omental thickening (arrows)

**Fig. 2:**
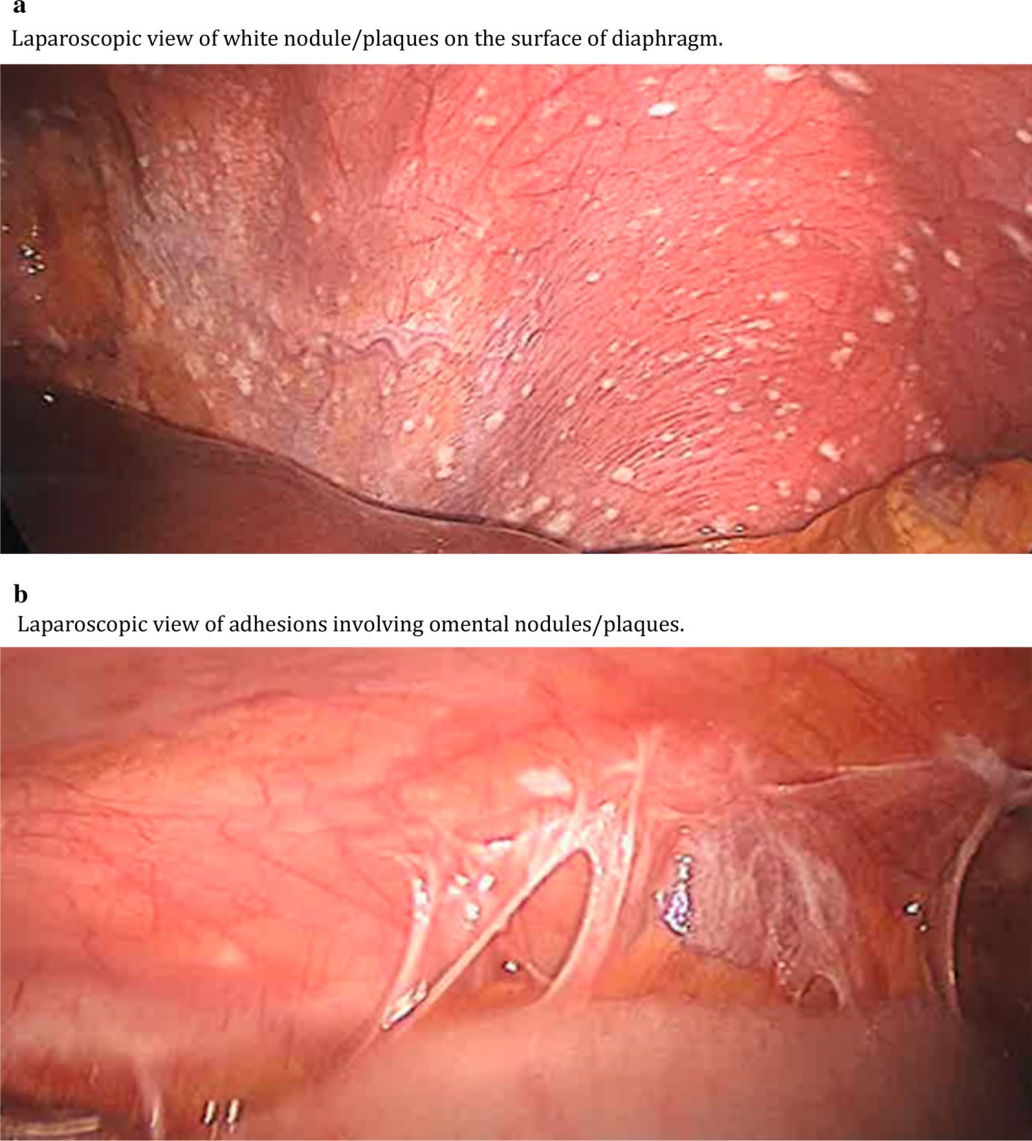
**a** Laparoscopic view of white nodule/plaques on the surface of diaphragm. **b** Laparoscopic view of adhesions involving omental nodules/plaques

**Fig. 3:**
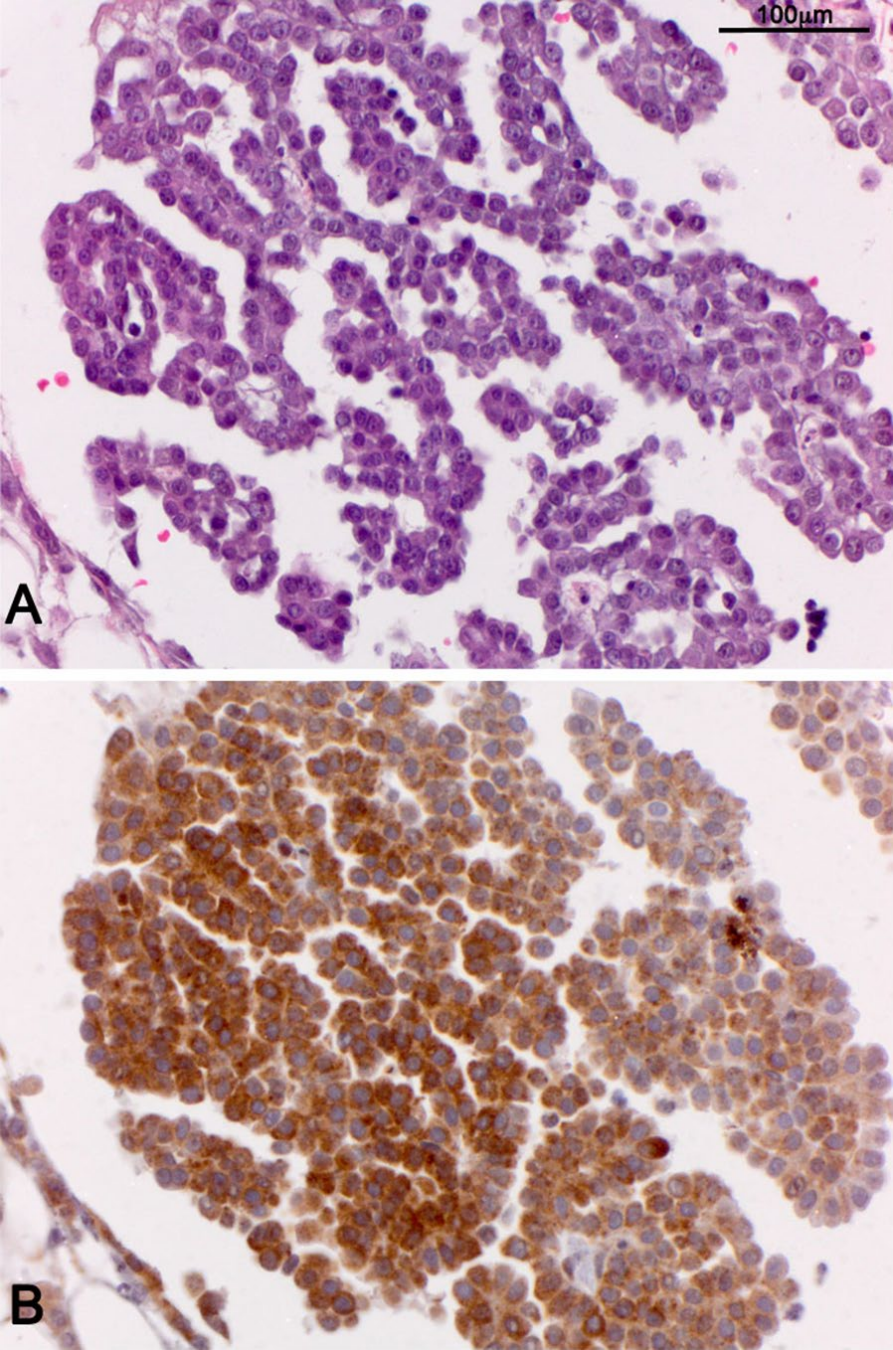
**A** H/E malignant mesothelioma, epithelial type. **B** BAP1 immunostaining. Note absence of nuclear staining indicative of biallelic BAP1 inactivation and accumulation of truncated inactive BAP1 protein the the cytoplasm. Note nuclear staining in few inflammatory cells infiltrating among tumor cells, as these cells retain one wild-type BAP1 allele. Original magnification ×200

A 45-year-old female presented with bilateral upper-quadrant abdominal pain. She is a non-smoker and had no known exposure to asbestos. Family history was significant for peritoneal mesothelioma in two family members—a sister who developed peritoneal mesothelioma at age 29, and a first degree male cousin who developed peritoneal mesothelioma at age 45. Physical examination revealed tenderness in the upper quadrants. Computerized tomography of the abdomen revealed omental stranding in the upper-quadrants (Fig. 1). Laparoscopy showed studding and multiple white plaques on the diaphragm and peritoneum with adhesions (Fig. 2a, b). Biopsies revealed epithelioid malignant mesothelioma (Fig. 3). Germline DNA was extracted from a blood sample, DNA extracted and BAP1 testing was performed by polymerase chain reaction followed by Sanger sequencing, as described [8, 9]. Genetic testing revealed that our patient carried the following inactivating truncating germline BAP1 mutation: chr3.52406884A > G, c.604T > C, p.Trp202Arg. This mutation inactivates the catalytic domain of BAP1, preventing autodeubiquitylation of the BAP1 protein and nuclear translocation [17].

She was treated with cytoreductive surgery, hyperthermic intraoperative peritoneal chemotherapy with cisplatin and doxorubicin, followed by adjuvant systemic chemotherapy, specifically six cycles of cisplatin and pemetrexed. She has been followed by clinical surveillance and utilizing diffusion weighted (DW)-MRI imaging every 6 months. She is doing well without any evidence of recurrence 24 months since treatment.

**Fig. 4:**
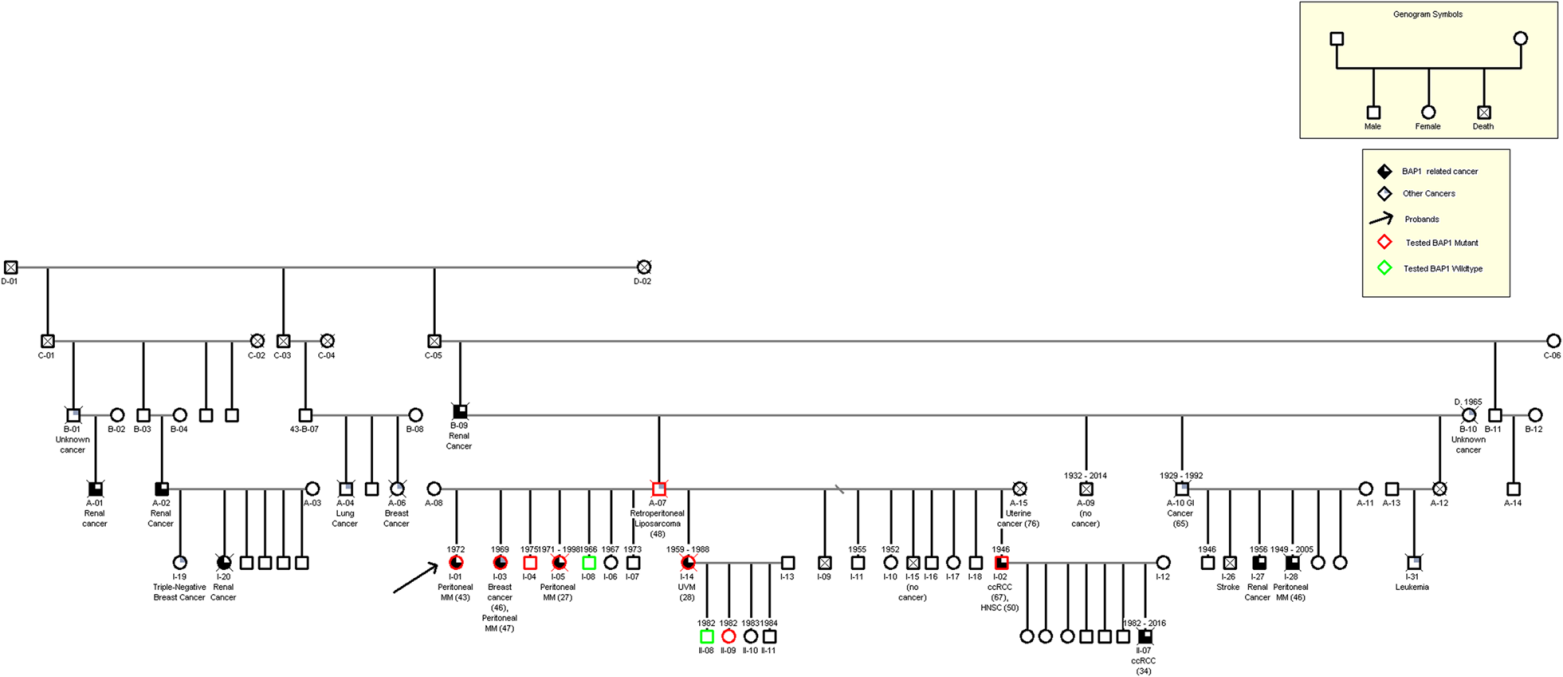
Family pedigree. Numbers above symbols represent year of birth and death, when available. Types of malignancies are listed below symbols and ages of diagnoses are indicated in parentheses, when available. Arrow indicates the proband; red symbols indicate individuals who were tested and found to be carriers of the BAP1 mutation; green symbols indicate individuals who were tested and found carrying wildtype BAP1 gene; *D* year of death, *MM* mesothelioma, *ccRCC* renal cell carcinoma, all other cancer types are indicated by their full name or anatomical location; “unknown”, the cause of death was cancer, but the histological type was not identified. “no cancer”, the cause of death was not cancer; A-07 was not tested for the presence of the BAP1 mutation, however he is an obligate carrier

The identification of a germline BAP1 mutation in this young lady with peritoneal mesothelioma, led to meetings with family members in which we explained the potential advantages and possible disadvantages of genetic testing for BAP1 mutations, consistent with the guidelines of the Consensus report on mesothelioma [7]. Eight family members elected to get tested: 6 were found to carry the same germline BAP1 mutation. The patient pedigree is illustrated in Fig. 4. Among them, 2 are tumor free and 1 has well differentiated papillary mesothelioma. Three of the carriers have other BAP1 related malignancies. Those who had not inherited the mutation were reassured that they did not carry a higher risk of developing mesothelioma or other cancers compared to the general population at large. Those who were found to carry germline BAP1 mutations, were informed of the increased risk for mesothelioma and other BAP1-associated cancers, and advised to reduce sun-exposure, minimize radiation exposure, including diagnostic/therapeutic radiation for the increased risk of cancer because of impaired DNA repair and apoptosis, and to avoid trades that could lead to asbestos exposure. In addition two of them had elected to enroll in a screening program, consisting of weighted (DW)-MRI imaging every 12 months utilizing diffusion weighted (DW)-MRI imaging rather than CT scans to avoid radiation exposure. All carriers are also undergoing annual detailed retinal exam by a retina specialist.

### Discussion

Carriers of germline BAP1 mutations have a very high incidence of cancer, most commonly uveal melanoma, malignant mesothelioma, cutaneous melanoma and clear cell renal cell carcinoma [1–6, 18, 19]. These malignancies occur at a younger age of onset than the general population. Inheritance is autosomal dominant with high penetrance. Other malignancies often reported in carriers of germline BAP1 mutations include most types of skin cancers [20–22], breast cancer, cholangiocarcinoma, non-small cell lung adenocarcinoma, meningioma, sarcomas, peripheral nerve sheath tumor, and neuroendocrine carcinoma [1–6, 9, 11, 23, 24].

Carriers of germline BAP1 mutations can also develop characteristic benign melanocytic skin lesions that were initially called as “atypical Spitz tumors” [25], and later when the unique histological and molecular characteristics of these lesions were elucidated, named as “melanocytic BAP1 mutated atypical intradermal tumors” (MBAITs) [6, 22].

Peritoneal and pleural mesotheliomas have both been associated with sporadic and germline BAP1 mutations [11, 24]. It has been estimated that only about 1% of mesotheliomas are associated with germline BAP1 mutations [7, 8, 10]. These mesotheliomas usually occur in patients younger than 55 and often without a history of asbestos exposure [9, 11]. This contrasts with the majority of pleural mesothelioma, which develop mostly among older people at an average age of 72 years. Most (70–90%) of these mesotheliomas are attributable to asbestos exposure [26, 27]. It is presently unclear if germline BAP1 mutations are sufficient per se to cause mesothelioma, as suggested by the spontaneous development of mesothelioma in mice carrying germline BAP1 mutations [28], or whether germline BAP1 mutations lower the threshold of asbestos exposure required to cause mesothelioma, as suggested by experiments in which lower amounts of asbestos were required to induce mesothelioma in heterozygous BAP1 mutant mice [29]. The possibility that germline BAP1 mutations increase susceptibility to low amounts of asbestos—amounts that would not be considered harmful for the population at large—is important, as rural areas continue to be developed exposing some cohort to asbestos and to other carcinogenic mineral fibers [30, 31]. Of note, asbestos is a term that was used for regulatory purposes to identify 6 different types of mineral fibers that were used commercially in the 1970’s. However, in nature there are over 300 additional mineral fibers that are potentially as carcinogenic as those called “asbestos” [32]. These fibers are not regulated and humans are increasingly exposed to them as rural areas are being developed [30–32]. Relatively low levels of carcinogenic fibers, as those found in areas with environmental exposure, might increase the risk of developing mesothelioma among carriers of germline BAP1 mutations [7].

The ratio of peritoneal to pleural mesothelioma is significantly higher in carriers of germline BAP1 mutations compared to the rate in the sporadic mesotheliomas [1]. In addition, in carriers of BAP1 mutations the majority of peritoneal mesothelioma occurs in women, and have a better prognosis, in contrast to sporadic mesotheliomas [4, 5, 11].

The critical role of normal levels BAP1 in preventing mesothelioma development is highlighted by multiple factors: (1) In families, BAP1 mutations specifically segregate among patients, while individuals who do not inherit the mutation are cancer free [5–9]; (2) tumors in carriers of germline BAP1 mutations always show loss of heterozygosity [5–9], i.e., biallelic BAP1 inactivation; (3) BAP1 is the most common acquired somatic mutation in sporadic mesothelioma [33–37].

Malignant mesothelioma or other BAP1 associated cancers occurring at a young age, i.e., 50 years old or younger, or occurring in multiple family members—regardless of age—should trigger testing for germline BAP1 mutations. This has important implications for the patient and for their relatives. Consensus guidelines suggest that screening for germline BAP1 mutations in mesothelioma patients who are members of families with multiple cases of mesothelioma, melanoma, renal cell carcinoma, cholangiocarcinoma, basal cell carcinoma, can lead to early detection that can be life saving for several of these malignancies, or at least to better therapeutic options when cancers are treated at an early stage [7].

## Conclusions

Studies in a remote region of Cappadocia led to the discovery that some families where uniquely susceptible to develop mesothelioma and that susceptibility was transmitted in a Mendelian fashion [38–40]. Those studies led to the discovery that inherited germline BAP1 mutations cause a high incidence of mesothelioma and of certain other cancer types in some families [5–9]. The challenge is to identify these cases of mesotheliomas because when mesothelioma and other cancers develop in carriers of germline BAP1 mutations, they often have a better prognosis. Moreover, management may be influenced by this information. For example, in our patient with BAP1 associated mesothelioma, monitoring for recurrence was accomplished using MRI rather than CT scans to reduce the risk of radiation-induced cancers. This risk cannot be underestimated in carriers of genetic mutations that impair DNA repair and apoptosis, as demonstrated in cancer patients affected by the Li-Fraumeni cancer syndrome who are also monitored preferentially by MRI [41, 42].

Moreover family members who inherited the same BAP1 mutations can benefit from prevention, screening and early detection [7]. Here we describe a case of malignant peritoneal mesothelioma occurring in a young lady with a strong family history of mesothelioma and no known asbestos exposure. These unusual findings—young age, family history, no history of exposure to asbestos made us suspect that the mesothelioma in our patient might have a genetic basis and that the patient might carry a germline BAP1 mutation. Testing confirmed our hypothesis. This finding led to genetic counseling for family members, and the identification of 6/8 who carried the same mutation. They have been informed about preventive measures to reduce the risk of cancer and are being followed for early detection that can be lifesaving. We feel our female patients early age of onset of peritoneal mesothelioma in the absence of known asbestos exposure occurred as a result of a genetic susceptibility. Whether a low level of environmental exposure to asbestos or other fibers [43] played a role is unclear.

Based on our experience we strongly recommend that mesothelioma occurring at a young age (< 50 years old), or in patients with multiple family members affected by mesothelioma or other cancers associated with germline BAP1 mutations should be tested for BAP1 gene mutations. Educating physicians about this rare disease is important. Early recognition of signs and symptoms of peritoneal mesothelioma in patients who carry germline BAP1 mutations can impact outcome and survival.

## Abbreviations

BAP1: BRCA-1 associated tumor protein 1
MBAITS: melanocytic BAP1 mutated atypical intradermal tumors
